# Innovative matrix for applying a food systems approach for developing interventions to address nutrient deficiencies in indigenous communities in India: a study protocol

**DOI:** 10.1186/s12889-019-6963-2

**Published:** 2019-07-15

**Authors:** Suparna Ghosh-Jerath, Shauna Downs, Archna Singh, Santanu Paramanik, Gail Goldberg, Jessica Fanzo

**Affiliations:** 1 Indian Institute of Public Health-Delhi, Public Health Foundation of India, Plot no 47, Sector 44, Institutional Area, Gurgaon, 122002 India; 2 Department of Health Systems and Policy, Rutgers School of Public Health, Rm. 426, 112 Paterson, St. New Brunswick, NJ 08901 Canada; 3 All India Institute of Medical Sciences, Ansari Nagar, New Delhi, 110029 India; 4 National Council of Applied Economic Research (NCAER), Parisita Bhawan, 11 Indraprastha Estate, New Delhi, 110002 India; 5 Nutrition and Bone Health Research Group, MRC Human Nutrition Research, Elsie Widdowson Laboratory, Fulbourn Road, Cambridge, CB1 9NL UK; 6 Department of International Health, Johns Hopkins Bloomberg School of Public, Health Berman Institute of Bioethics, Johns Hopkins Nitze School of Advanced International Studies, 1809 Ashland Avenue, Baltimore, MD 21205 USA

**Keywords:** Food systems research, Indigenous foods, Indigenous communities, Nutritional deficiencies

## Abstract

**Background:**

Indigenous communities retain knowledge of the land and food resources rooted in historical continuity within their region of residence. Food systems research can be leveraged to identify strategies to encourage sustainable use of complex multi-species agroforestry systems by indigenous communities contributing to nutritional needs while simultaneously preserving the ecosystems and their benefits to society. Till date, the analyses of food systems have predominantly focused on high income countries often overlooking the alternatives (dietary and production) that would be most relevant to low and middle income countries (LMIC). Thus, innovative methodological approaches are needed to comprehensively characterize diverse food systems in LMICs with special reference to indigenous communities.

**Design:**

This protocol paper describes a food systems approach that will be employed to understand diverse and dynamic food systems of vulnerable tribal communities of Jharkhand, India and leverage their agroforestry systems to improve dietary diversity, nutrition status and address food security. Four tribal groups namely Santhal, Ho, Munda and Sauria Paharia of Godda, West Singhbhum and Khunti districts of Jharkhand would be studied.

This will be an exploratory cross-sectional study design, along with a longitudinal component to capture seasonality in dietary intake and agricultural diversity. A mixed methods approach will be used based on a conceptual framework on drivers of food systems, food supply chain, food environment (both wild & cultivated, and market food environments), as well as consumer behaviour and maternal and child health outcomes in tribal communities. The quantitative surveys will be conducted on socio-economic, demographic profile of households, their availability of, access to and utilization of food environment and nutritional status of reproductive age group women and children under 5 years. Qualitative enquiries will examine barriers and facilitators to increase sustainable production, procurement and consumption of indigenous foods. The final outcome would be development of interventions to promote indigenous food consumption.

**Discussion:**

By utilizing a combination of value chain analysis and ‘Optifoods linear programming software’ that will use above information on indigenous community, dietary intake, nutritional status and food environment, evidence based interventions promoting indigenous food systems aimed at addressing food and nutritional security of tribal communities will be developed.

## Background

The central paradigm of survival is the maintenance of the integral synergism between humans and their environment. While modern agricultural practices to improve food sufficiency have increased agricultural productivity, they have also put a strain on the environment. Food production (and the way it moves along the value chain) that primarily focuses on mono-cropping makes diets less diverse, alongside contributing to biodiversity loss and environmental degradation. As such it is associated with large carbon and water footprints making our current food system unsustainable [1–3] Despite an increase in the productivity of major food crops, the number of people around the world who are food and nutrition insecure remains high, with many countries battling with multiple burdens of malnutrition. On one hand, 821 million people are undernourished, over 50 million children under five are wasted and 770 million people have severe food insecurity. Further, 151 million children under five years of age are stunted and approximately two billion people worldwide have micronutrient deficiencies. Coinciding with the burden of undernutrition, is the increasing prevalence of overweight and obesity as well as diet-related non-communicable diseases (e.g., diabetes, cardiovascular disease, etc.) [4–6]

Most of the world’s food insecure people live and work in rural areas – small-scale subsistence farmers still constitute 50% of rural populations in developing countries [7–9]. Indigenous and tribal people who rely on subsistence farming, are descents from populations, who inhabited the country or geographical region at the time of conquest, colonisation or establishment of present state boundaries and have their own cultures, languages, customs and institutions. In addition to that and importantly, they retain knowledge of the land and food resources rooted in historical continuity within their region of residence. [10, 11].

Nevertheless, many subsistence crop production systems of these indigenous communities are characterized by low productivity and instability of production often attributed to marginal and erratic rainfall. [12, 13]. However, globally we also have scenarios where indigenous knowledge has enabled the communities to enhance subsistence farming at the time of seasonal and climatic variability. This is often referred in literature as ‘community based adaptation’ defined as community led processes based on community’s priorities, needs, knowledge and capacities which have empowered people to plan and cope with the impacts of climate change [4, 14, 15].

Food systems research can be leveraged to help understand and explore these indigenous food systems and analyse the gaps in food production and challenges that hinder access and optimum utilization by these vulnerable indigenous populations. More specifically, strategies can be identified that can encourage the sustainable use of complex multi-species agroforestry systems (AFS) (see Table 1) by these indigenous communities that can contribute to meeting nutritional needs while simultaneously preserving the ecosystems and its benefits to society [19, 20].

**Table 1:** An overview of Agroforestry Systems (AFS)

Definition	AFS combines wild and domesticated plants, animals, fungal and micro-organic components as well as their interactions. It also includes traditional species of foraged and cultivated floras and faunas that are adapted to the local geographical, climatic and growth conditions [16].
Significance	AFSs are designed and managed based on millenarian experiences of people throughout the world and are expressions of their traditional ecological knowledge and biocultural heritage [17, 18], of which indigenous communities are important custodians [9].
AFS for nutrition intervention	The usage of AFS by indigenous communities make it a potentially important strategy for providing low resource intensive, nutrient rich and sustainable sustenance [7].

Despite the traditional ecological knowledge (TEK) and availability of rich AFS, indigenous communities are often vulnerable to nutritional deficiencies and food insecurity [21]. Since AFS biodiversity does not necessarily translate into dietary diversity and food security of these communities, ways are needed to leverage the potential of AFS by contextualizing associations among agricultural diversity that exists within subsistence farming systems, dietary diversity, nutritional status and food security [22].

Thus, to improve sustainability and support a healthier and nutritious food supply in vulnerable indigenous communities, [7, 23] suitable frameworks for identifying strategies to promote utilization of indigenous food resources are required. Till date, the analyses of sustainable diets and food systems have predominantly focused on high income countries often overlooking the alternatives (dietary and production) that would be most relevant to LMICs. Thus, innovative methodological approaches are needed to comprehensively characterize diverse food systems in LMICs with special reference to indigenous communities. [24].

The food system encompasses the elements and activities that relate to the way in which food is produced, processed, distributed, prepared and consumed, and the outcomes of those activities in terms of nutrition, health, equity and the environment [25]. In order to ensure that populations worldwide have access to culturally appropriate food that meets their nutritional needs, food systems need to be leveraged in order to improve the quality of food that is produced, moves along the food supply chain, and is available, affordable, safe and desirable within food environments. By applying a food systems approach for examining the nutritional challenges faced by indigenous populations in LMICs, there is greater potential to identify upstream drivers of food choice and potential solutions across sectors to confront the challenges of improving nutrition and health, while simultaneously protecting the environment [24]. Further, it is important to document the spectrum of ecosystem management, roles of agroforestry, TEK and human culture for food sovereignty in LMIC set up through a defined approach [26].

Our present study, will employ a food systems approach to understand the diverse and dynamic food systems of vulnerable tribal communities of Jharkhand, India and leverage their AFS to improve dietary diversity, nutrition status and address food security. This will allow us to gain a thorough understanding of the food system as a whole, the multiple synergistic factors and drivers of food choices and to identify points of intervention throughout the food system to support good nutrition among these vulnerable communities. This will culminate in the development of an innovative matrix to analyze indigenous food systems in the LMIC context.

## Methods/design

### Study setting and population

Despite bountiful natural resources, Jharkhand, a state in the central eastern part of India is amongst the poor performing states with one of the highest burdens of malnutrition. The state has a large indigenous population (26.2%), known as the Scheduled Tribes (ST) with a total population of the state being 32 million [27]. Out of thirty of such Scheduled Tribes in the State, the Santhal are the most populous constituting 34% of the state’s total indigenous population. The Oraon (19.6%), Munda (14.8%) and Ho (10.5%) tribes also make up a large proportion of the total ST population of the State. Sauria Paharias (less than 1%) are one of the primitive tribal groups in Jharkhand (and are considered as particularly vulnerable) [27]. Our study will focus on the Santhals, Ho and Munda tribes of Jharkhand as well as the Sauria Pahariya tribes given their vulnerability. The study districts would include Godda, West Singbhum and Khunti as they have high concentration of the specific tribal groups under study.

### Overview of conceptual framework

**Fig. 1:**
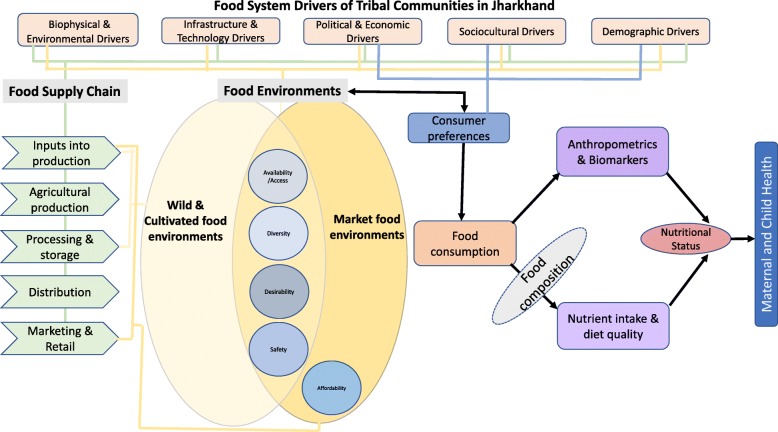
The conceptual framework of the characterization of the food system in Jharkhand, India

Figure 1 depicts our project’s conceptual framework, which describes the drivers of the food system, the food supply chain, the food environment (both wild and cultivated, and market food environments), as well as consumer behaviour and maternal and child health outcomes in tribal communities in Jharkhand. We adapted the conceptual framework of the food systems from the High Level Panel of Experts (HLPE) report on Nutrition and Food Systems, [25] to develop our framework to better reflect our study population and design.

The drivers of food systems in tribal communities in Jharkhand feed into the food supply chain and food environments which then influence the availability, diversity, desirability, safety and affordability of foods. These factors, along with consumer preferences, affect the foods that these communities acquire and consume. The nutrient composition of consumed foods has significant implications in terms of nutrient intakes and diet quality, body composition and micronutrient status, thus influencing nutritional status and maternal and child health outcomes.

## Aim of the study

The overarching aim of this study is to evaluate the potential of indigenous foods in contributing to dietary diversity and nutrient intake for improving food security and nutritional status of vulnerable tribal communities of Jharkhand, India. The specific objectives of this study, target four main areas: 1) characterizing the food environment; 2) assessing the nutritive value of indigenous foods that are routinely accessed; 3) estimating the contribution of indigenous foods to nutrient intake and nutritional status of the communities; and 4) informing the development of interventions to support indigenous food consumption.

### Overarching study design

We will use an exploratory cross-sectional study design, along with a longitudinal component to capture seasonality, in order to examine the food system of tribal communities in Jharkhand.

### Study procedures

A mixed methods approach will be used for addressing each of the four objectives of the study.

**Fig. 2:**
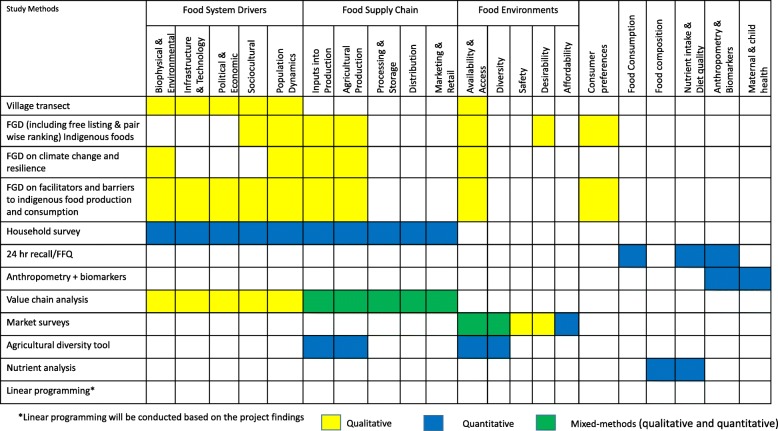
Overview of the different methods to be used for assessing different components of the conceptual framework

Figure 2 provides an overview of the different methods that will be used as part of this study and for assessing different components of the conceptual framework addressing each objective of the study. We describe these methods in additional detail below.

### Overall sample

A two-stage cluster sampling design will be adopted for each of the tribal groups. Although there are some variations in the sampling strategy across tribal groups, the general methodology consists of first randomly selecting villages (from purposively chosen blocks based on the population density of tribal groups under investigation) using probability proportional to size (PPS) sampling, with size being the number of households in the village as per census 2011. Villages with less than 50 households will be excluded, as the required number of eligible households (having at least one child 6–59 months of age) may not be available. In the second stage, a house-listing exercise will be carried out in all sampled villages in order to construct the sampling frame of eligible households, as described in additional detail below. Under the assumption of needing at least 20 eligible households per village, we plan to select 14 villages (from purposively chosen blocks; a block is defined as an administrative sub division of a district) for each of the four tribal groups; Santhal, Ho, Munda and Sauria Paharia. For each tribe, the total number of villages (*n* = 14) will be distributed approximately equally to selected blocks. Number of blocks would vary across tribes depending on the area of habitation of the tribe in respective district. Household information will be elicited from any adult member of the household. The dietary recall and anthropometric measures would be taken on one eligible woman in the reproductive age group and an infant or child (between 6 months to 5 years) in each eligible household. In addition to this, biochemical assessment using finger prick blood samples will be done on the selected woman.

#### Aim 1. Characterizing the food environment

Aim 1 of the study involves characterizing the food environment, including the cultivated, wild and the market environments that the indigenous populations included in the study interface with. As part of this aim, we will address the following objectives: 1) appreciate knowledge and perception of specific tribal communities regarding their use of indigenous foods 2) map the biodiversity and assess availability, access and utilization of indigenous foods; 3) assess any shift in dietary patterns vis-à-vis indigenous food intake; 4) characterize farming systems to understand constraints and opportunities for improving indigenous food production and the resilience of these systems to climate variability; and 5) examine barriers and facilitators to increase sustainable production, procurement and consumption of indigenous foods. Table 2 provides an overview of the specific methodological approach that will be used to address each of the sub-objectives under aim 1 and their anticipated outcomes.

**Table 2:** Summary of the methodological approaches, tools used and variables captured for objective 1

Objectives and sub objectives	Methodological approaches and activities	Tools used	Source for primary data Or secondary data	Variables captured
Characterize the food environment				
Appreciate knowledge and perception of specific tribal communities regarding their use of indigenous foods Assess any shift in dietary patterns in these communities vis-à-vis indigenous food intake	Gathering traditional food list data ✓ Traditional food seasonality and popularity in the community among various age groups ✓ Little used or currently unused traditional foods known to elders ✓ Developing traditional food list ✓ Selecting short list of potential micronutrient rich foods for more detailed study ✓ Patterns of harvest, storage and preparation ✓ Identifying shifts in dietary patterns (if any) and perceptions regarding indigenous foods	PRA exercises using qualitative tools Focus group discussions, in-depth interviews Study tools adapted from CINE (Centre for Indigenous Peoples' Nutrition and Enviroment) protocol [28]	Community, adults, elders, traditional healers, community workers Secondary data on published literature, reports, documents	List of indigenous foods List of indigenous foods, not used currently Potential micronutrient rich indigenous foods Information on harvest, storage and preparation Information on present vs previous diets
Map the biodiversity and assess availability, access and utilization of indigenous foods by these communities	Village transect to understand the social, physical and ecological characteristics of the community and its surrounding area Village map	Village transect question guide Village map	Group of village adults (men and women) who know the territory well e.g. village elders, religious leaders, community leaders, frontline workers like anganwadi workers and Accredited Social Health Activists (ASHA)	Infrastructure like kind of households, electricity, road; irrigation facility and water sources, types of trees, useful plants, location for productive activity like agriculture, livestock, human settlements, type of vegetation, type of soil, plots, types of crops, grazing and forest land, visible problems that may affect food security, schools, shops, markets, places of worship, medical facility Location of the social and ecological characteristics, natural and physical resources, neighborhoods within the community, vulnerable households, patters of access and control over resources for food security.
Characterize farming systems to understand constraints and opportunities for improving indigenous food production and Resilience of present farming system to climatic variability	Agricultural diversity Food from the market ✓ Historical timelines and climate trends ✓ Changing farming practices and crop ranking ✓ Climate risk and coping mechanism	Household survey (tab based questionnaire for electronic data capture) Market survey Qualitative Focus groups discussions and key informant interviews Climate risk and coping mechanism matrix	Information from households on farming system/agricultural practices, crops species grown in the agricultural fields and backyards, products collected from forests Vendors and shop owners, community members Men and women of all ages, include elderly members	Agricultural diversity calculated from MFAD and Shannon entropy index for the region Crop species richness in the tribal communities Present use of indigenous varieties vs hybrid varieties Present products in the market, accessibility to the community, cost and affordability of nutrient rich food items Main climate events in the past, climate tendencies over the years, periods of food insecurity or even famine. Impact on livelihood strategies, natural resources and food systems. Changes in farming practices, their causes and consequences, impact of changes in farming practices, the impact of changes in food security, main characteristics of indigenous crops. Types of climate risk, consequences of climate risk and the existing adaptive capacity
Examine barriers and facilitators to increase sustainable production, procurement and consumption of indigenous foods		FGDs with community members, community leaders	Men and women in the community	Types of products (food and non-food items) collected, livelihood strategies (including the intensification of subsistence production), beliefs Types of products (food and non-food items) cultivated, grown (land, kitchen garden, ponds) Identify livelihood strategies, including the intensification of subsistence production Beliefs, perceptions and practices on foraging or hunting Traditional and legal rights to access forest areas Seasonality of forest products (food and non-food items) Traditional roles of foraging/hunting Transition into market economy- emerging market-based opportunities for forest products Access to food items sold in the market, or community stores Access to food items from government schemes like PDS (Public Distribution System (PDS), Anganwadi centres

We will use tools developed by the Centre of Indigenous Peoples’ Environment and Nutrition [28] as well as a toolkit developed by Bioversity International and the Institute of Development Studies on climate change and food security vulnerability [29] to guide our methodological approach for characterizing the food environment. We will use a combination of a village transect, semi-structured interviews (including free listing) and focus group discussions to: assess knowledge and perception of tribal communities regarding indigenous foods, map biodiversity, identify commonly consumed indigenous foods, assess shifts in dietary pattern, examine resilience of the present farming systems to climate variability and barriers and facilitators to sustainable production and consumption of indigenous foods. These qualitative methods will be complemented with quantitative methods that include an agricultural diversity tool for assessing crop species richness [24] (e.g., Shannon diversity, Modified Functional Attribute Diversity (MFAD) and population share with adequate nutrients) and a market survey that will provide insight into the price and availability of foods in the market food environments that the tribal communities have access to, so that we can examine the affordability and diversity of foods in the markets and identify gaps in food market access. The market survey has been adapted from a tool previously used to examine markets in Myanmar [30].

#### Data analysis

FGDs and interviews will be digitally recorded and transcribed verbatim. The FGD and interview transcripts will be coded in Atlas ti using grounded theory, based on the emergence of themes. We will use open, axial and selective coding to organize our data by key themes relating to the production and consumption of indigenous foods as well as the broader food system. The data analysis will be iterative and will continue to the point of theoretical saturation.

For assessing agricultural biodiversity, three separate measures namely crop species richness, crop varietal richness and crop nutritional functional richness will be calculated. [31, 32]

#### Aim 2. Assessing the nutritive value of indigenous foods

In order to assess the nutritive value of commonly consumed indigenous foods, standard protocols will be used to prepare herbariums of collected plant foods [33], followed by taxonomic classification by a botanist. After the identification, existing food composition tables will be searched to identify pre-existing information on the nutritive value of these foods, otherwise the item will be sent for nutrient analysis. Required quantities of the food sample for analysis will be collected from field sites, and transported in ice-coolers to the laboratory. The nutrient analysis will be carried out by a NABL (National Accreditation Board for Testing and Calibration Laboratories) accredited laboratory after verification of methods and quality check procedures. The sample collection and nutrient analysis will be done according to standard reference protocols developed as part of the study in consultation with the laboratory. The gravimetric method will be used for energy and fat, titrimetric for protein while carbohydrate will be measured by difference. The vitamins will be assessed using High Performance liquid Chromatography (HPLC) and the minerals by Inductively Coupled Plasma Mass Spectrometry (ICP-MS). The analyte values will be reported per 100 g of edible weight. The quality control procedures will be assessed, supervised and documented by the core research team throughout the analytical process. [34]

#### Aim 3. Estimating the contribution of indigenous foods to nutrient intake and nutritional status

The contribution of indigenous foods to the nutrient intake of the communities in our study, will be estimated through household surveys including dietary intake surveys and assessments of nutritional status, described in detail below.

##### Sampling strategy

All eligible households will be selected from the sampled villages (as explained in the overall sample section). A list of eligible women and children (6–23 months and 24–54 months) in each household will be prepared. We plan to have two strata of sample households. The first stratum will include children in the age range of 6–23 months and the second stratum will have children 24 to 59 months. In a household with a child from both the strata, the child in the first stratum will be selected. If there is more than one child in the same stratum, a Kish table will be used to select which child will be included in the study sample. One eligible woman from the selected household will then be randomly selected using a Kish table [35]. Note that the selected woman and the selected child’s mother may be two different respondents in a specific household. We will assess dietary intakes and anthropometric measurements in the selected women and children, as described below.

##### Sample size

We based our sample size calculation for each tribal population on the difference in mean dietary intake of iron (mg/day) in women who consumed indigenous food as compared to women who did not consume indigenous food. Our sample size calculation suggests that we need at least 134 women per group in each of the tribal communities (leading to a total of 268 households per tribal group) to detect a difference of at least 30% in iron intake (> 3.4 mg difference in absolute terms) between the two groups with 80% statistical power based on a two-sided test having 5% level of significance, after accounting for the loss in precision due to cluster sampling (assuming a design effect of 2). To calculate our sample size, we used estimates of mean iron (11.3 ± 7 mg/day) in the Santhal tribes based on previously published work [36]. In order to ensure that we have a large enough sample size, we plan to include 280 households per tribal group (280 × 4 tribal groups = 1120 households).

##### Households surveys

An interviewer administered questionnaire will be used for the household surveys. Information will be collected on socio-economic and demographic characteristics, dietary intakes of women and children using a 24 h dietary recall and food frequency questionnaire (FFQ), traditional food patterns, seasonal dietary habits, procurement of food, food expenditure, infant and young child feeding practices of the index child and household food security. Anthropometric and biochemical assessments will be done in women and children. Data will be collected in real time on a tablet using CSPRo software (version 7) with inbuilt range checks.

##### Food security and dietary intake data

Household food security will be assessed using the Food Insecurity Experience Scale which consists of eight questions related to the quantity and quality of foods that households have access to (FAO) [37]. The frequency of consumption of foods at the household level including indigenous foods will be assessed using a Food Frequency Questionnaire (FFQ). The FFQ will be based on a list of indigenous food items compiled from foods identified during qualitative enquires (i.e., free listing) conducted in specific tribal groups.

In addition to the FFQ, 24 h dietary recalls will be taken on two non-consecutive days for each selected woman and child per household. The woman respondent will be asked by an interviewer to recall, in as much detail as possible, her own food intake or the intake of her child during the past 24 h using a food recall kit which includes measuring cups, spoons and a portion size flip book. The respondent will be asked to recall, and wherever possible show or describe the foods eaten in each meal (i.e. each food item consumed along with a detailed recall of ingredients used, method of preparation, etc.). The recall will be repeated in another season on one third of the study participants to account for variability in seasonal nutrient intake. A digital pen technology will be used for documenting dietary recalls and FFQs. The data collection will happen in real time and the data entered in the 24 h dietary recall sheets will be directly entered into a database that will be linked to the data from Indian food composition table [38] and other primary and secondary nutrient databases. The 24 h dietary recalls will also be used to assess the Minimum Dietary Diversity – Women (MDD-W) indicator, [39] Minimum Dietary Diversity, minimum meal frequency and minimum acceptable diet indicators will be used to assess dietary diversity in children.

##### Anthropometric assessments

Anthropometric assessments will be carried out on the same women and children for whom the dietary recall has been documented. Anthropometric measurements such as weight, height will be taken using standard protocols [40] and equipment. In children, Mid-upper Arm Circumference (MUAC) will also be measured. Body mass index (BMI) will be calculated as weight (kg)/ height (in meters^2^) for women. The women will be classified as underweight or otherwise, using the BMI cut-offs [41]. The anthropometric assessment data from children will be entered in World Health Organization (WHO) “Anthro” software (version 3.2.2, 2011). Percent distribution of children according to weight for age, height/length for age, weight for height/length, as per WHO classification [42] will be conducted in order to assess prevalence of stunting, wasting and underweight.

##### Biochemical assessment

Five analytes (micronutrient and inflammatory biomarkers) will be concurrently estimated in capillary blood samples from 280 female subjects of reproductive age, from two tribal groups. These will be the same women on whom 24 h DR and anthropometric assessments will be done. The analytes include α- acid glycoprotein (AGP), C-Reactive Protein (CRP), ferritin, Retinol Binding Protein (RBP) and soluble Transferrin Receptor (sTfR). Samples will be collected from the selected woman in each household, by a certified, experienced laboratory personnel based on previously framed standard operating procedures. The samples will be stored after centrifugation and serum separation, in cryovials in a freezer at the field site and transported to the laboratory after collection is complete in one tribal group. The estimation of the biomarkers will be done using the Luminex assay™ with drop array technology. Assay characteristics for all analytes, such as the Limit of Detection, Limit of Quantitation, dynamic range and linearity across concentrations will be defined and validated prior to estimating the biomarkers in the participants’ serum. The biomarker concentrations will be adjusted for inflammation using the CRP and AGP before reporting the final values.

All the study tools will be translated by a native speaker into the local language and back-translated into English to check for fidelity, accuracy, and consistency. All data on HH survey, dietary intake, anthropometric measurements will be collected by field investigators and nutritionist after providing due trainings. The field investigators will be selected from the local communities, who are well versed with the local language. Core team members will always accompany the data collection team and will do periodic checks on the collected data.

##### Data analysis

Nutrient intake data will be compared with Recommended Dietary Allowances (RDA) for Indians [43] for a moderately active adult female and for children under five and with WHO recommendations for complementary feeding for breast fed and non-breast fed children [44]. The adequacy of nutrient intake will be computed as Nutrient Adequacy Ratios (NAR) [45]. The NAR is equivalent to an individual’s nutrient intake of a day divided by the RDA for the respective nutrient. The cut offs for the NAR are: inadequate (< 0.66), fairly adequate (0.66- < 1.00) and adequate (≥1.00).

Continuous variables will be summarized as mean ± standard deviation (SD) or as median and interquartile range (IQR), while categorical variables as number of subjects and percentages.

Women and children will be categorized into groups consuming and not consuming indigenous food based on the 24 h dietary recall. The nutrient intake, nutritional status and biomarkers in the two groups will be then compared. The t-test or Wilcoxon Rank sum test and chi-square test will be used to compare the differences in distribution of continuous and categorical variables, respectively. Two-sided tests will be used with statistical significance set at *P* < 0.05. Analyses will be performed using STATA 13 (StataCorp, College Station, USA).

#### Aim 4. Informing the development of interventions to support indigenous food consumption

##### Value chain analysis and linear programming

We will conduct a combination of value chain analysis and linear programming to help identify potential interventions aimed at supporting indigenous food consumption. More specifically, value chain analysis of the indigenous foods that have the greatest potential to address the nutrient gaps identified in Aim 3 will be conducted. The ways in which the selected indigenous foods move through the value chain from field-to-fork will be mapped with the view to identifying the bottlenecks in the value chain, and potential interventions that could improve their availability, affordability and acceptability. The value chain analysis will use a combination of semi-structured interviews (n = ~ 30) with purposively selected value chain actors and market analyses. The interviews will include topics related to the production of indigenous foods (including use of inputs and yields) as well as their processing, storage, retail (including profitability) and preparation. We will triangulate these data with data from the focus group discussions examining the barriers and facilitators to indigenous food consumption from Aim 1.

Additionally, we will use the linear programming software "Optifood" to inform the development of food-based recommendations based on the nutrients that tribal communities can obtain from their local food environments. Optifood analyzes the dietary patterns of target groups, and the costs of local foods, to identify the lowest cost diet that will meet the nutrient needs of the target group. We will use a combination of the dietary data from Aim 3 and the market data from Aim 2 as the basis for the Optifood analysis. [46].

## Discussion

This study will provide the first comprehensive examination of the food system of tribal communities in Jharkhand state. Our exhaustive examination of the different components of the food system, and the way in which communities’ interface with them, will facilitate development of context-specific interventions that are tailored to the needs of the population. Through our findings, we will identify interventions to help support the sustainable production and consumption of indigenous foods targeted at specific stakeholder groups. These interventions would ideally be incorporated into existing strategies and programs and help achieve the ultimate goal of the project which is to address food security, undernutrition and better nutrient intake in women and children as well as facilitate ecosystem conservation and biodiversity preservation.

In addition to informing the development of interventions to address the burden of malnutrition in the tribal communities in Jharkhand, the study will also build a methodological foundation for the development of future studies that aim to conduct a comprehensive assessment of the food systems of local communities. There is a dearth of empirical research examining local food systems and little is known about the best approaches to measuring them. This research will help contribute to the existing evidence base of measuring food systems in LMIC contexts.

A prototype for a multi-level approach including development of an evidence-based, context specific package of nutrition-sensitive interventions for encouraging production and consumption of these indigenous foods will be the primary outcome of the study. The prototype intends to inform how to incentivize production and consumption of indigenous foods throughout the food system as a whole. Finally, the robust scientific content would help in creating a conducive atmosphere for acceptance of indigenous foods as a bona fide and sustainable nutritional enhancement strategy in this part of the world.

## Abbreviations

AGP: α- acid glycoprotein
AKS: Agroforestry Systems
BMI: Body Mass Index
CRP: C-Reactive Protein
FFQ: Food Frequency Questionnaire
FGD: Focus Group Discussions
HLPE: High Level Panel of Experts
HPLC: High Performance liquid Chromatography
ICP-MS: Inductively Coupled Plasma Mass Spectrometry
LMIC: Low and Middle Income Countries
MDD-W: Minimum Dietary Diversity – Women
MFAD: Modified Functional Attribute Diversity
MUAC: Mid-upper Arm Circumference
NABL: National Accreditation Board for Testing and Calibration Laboratories
NAR: Nutrient Adequacy Ratios
PPS: Probability Proportional to Size
RBP: Retinol Binding Protein
RDA: Recommneded Dietary Allowances
sTfR: soluble Transferrin Receptor
TEK: Traditional Ecological Knowledge
